# Securing China’s rice harvest: unveiling dominant factors in production using multi-source data and hybrid machine learning models

**DOI:** 10.1038/s41598-024-64269-0

**Published:** 2024-06-26

**Authors:** Ali Mokhtar, Hongming He, Mohsen Nabil, Saber Kouadri, Ali Salem, Ahmed Elbeltagi

**Affiliations:** 1 School of Geographic Sciences, East China Normal University, Shanghai, 210062 China; 2 Department of Agricultural Engineering, Faculty of Agriculture, Cairo University, Giza, 12613 Egypt; 3 Division of Agriculture Applications, Soils, and Marine (AASMD), National Authority for Remote Sensing and Space Sciences (NARSS), Cairo, Egypt; 4 Laboratory of Water and Environment Engineering in Sahara Milieu (GEEMS), Department of Civil Engineering and Hydraulics, Faculty of Applied Sciences, University of Kasdi Merbah Ouargla, PB 147 RP, 30000 Ouargla, Algeria; 5 Civil Engineering Department, Faculty of Engineering, Minia University, Minia, 61111 Egypt; 6 Structural Diagnostics and Analysis Research Group, Faculty of Engineering and Information Technology, University of Pécs, Boszorkány ut 2, H-7624 Pecs, Hungary; 7 Agricultural Engineering Depeartment, Faculty of Agriculture, Mansoura University, Mansoura, 35516 Egypt

**Keywords:** Climate change, Vegetation indices, Food security, Hybrid machine learning models, Rice production, Mathematics and computing, Climate change, Plant sciences

## Abstract

Ensuring the security of China’s rice harvest is imperative for sustainable food production. The existing study addresses a critical need by employing a comprehensive approach that integrates multi-source data, including climate, remote sensing, soil properties and agricultural statistics from 2000 to 2017. The research evaluates six artificial intelligence (AI) models including machine learning (ML), deep learning (DL) models and their hybridization to predict rice production across China, particularly focusing on the main rice cultivation areas. These models were random forest (RF), extreme gradient boosting (XGB), conventional neural network (CNN) and long short-term memory (LSTM), and the hybridization of RF with XGB and CNN with LSTM based on eleven combinations (scenarios) of input variables. The main results identify that hybrid models have performed better than single models. As well, the best scenario was recorded in scenarios 8 (soil variables and sown area) and 11 (all variables) based on the RF-XGB by decreasing the root mean square error (RMSE) by 38% and 31% respectively. Further, in both scenarios, RF-XGB generated a high correlation coefficient (R^2^) of 0.97 in comparison with other developed models. Moreover, the soil properties contribute as the predominant factors influencing rice production, exerting an 87% and 53% impact in east and southeast China, respectively. Additionally, it observes a yearly increase of 0.16 °C and 0.19 °C in maximum and minimum temperatures (T_max_ and T_min_), coupled with a 20 mm/year decrease in precipitation decline a 2.23% reduction in rice production as average during the study period in southeast China region. This research provides valuable insights into the dynamic interplay of environmental factors affecting China’s rice production, informing strategic measures to enhance food security in the face of evolving climatic conditions.

## Introduction

Early and accurate crop production forecasting is essential for policymakers to make timely decisions for export–import commerce, which is the foundation for a country's food security^1^. It is also necessary for agricultural producers to avoid bad crop selection, which could cause incalculable losses in profits due to over-production and under-production^2–4^. Moreover, the cropland loss observed in various nations over the past years with high food demand owing to population growth requires accurate and up-to-date crop yield forecasting to maintain food security^5^. To prevent these losses, predicting crop production is required. However, human predictions are not effective with increasing amounts of agricultural data. Instead, machine learning has been raised as a promising option for this goal^6^.

Machine learning was created in data mining as a methodology for teaching computer concepts^7–11^. This model uses the learning idea to predict new sets of data given big data sets through training and testing. The present study selected rice as one of the world's three major crops extensively farmed and consumed, along with wheat and maize^12–14^. Nearly 88% of the world's rice is grown in Asian nations, where 2.4 billion people eat rice daily^15^.

Given the importance of rice to national food security, several studies implemented various machine-learning techniques for forecasting rice yield. Jabjone and Jiamrum^16^ developed an artificial neural network (ANN) model to predict rice production in the Phimai district, Thailand. The developed ANN model achieved highly accurate estimation with low errors (low RMSE) in rice yield forecasting using meteorological factors, including rainfall, water distribution, evapotranspiration, temperature, humidity, and wind speed^16^. Marndi, Ramesh^17^ applied long short-term memory (LSTM) for predicting rice yield using different input scenarios. The best LSTM model was achieved using rainfall as an input variable for rice yield forecasting. Sultana and Khanam^18^ compared the performance of Auto-regressive Integrated Moving Average (ARIMA) and Artificial Neural Network (ANN) on univariate time series data of yearly rice production from 1972 to 2013. According to this study, the ARIMA model outperforms the ANN model since the estimated error of ANN was significantly higher than ARIMA errors. In addition, Balakrishnan and Muthukumarasamy^1^ suggested an ensemble model to predict crop production over time based on the Ada support vector machine (SVM) and Ada and Naive Bayes (Naive), where Ada SVM and Ada Naive performed better than SVM and Naive Bayes.

Multiple input variables were used in rice yield estimation, including climatic data, remote sensing data, and statistical data (e.g. sowing area). Climatic variables showed a significant relationship with rice yield in several studies^16,17,19,20^. For example, the temperature increases by 1–2 °C during the paddy earring stage causing a decrease in paddy rice production by 10–20%^21^. Compared to technology, input, and social and economic factors, climate factors individually explain 84% of the variation in paddy rice production^22^. Moreover, remote sensing vegetation indices such as normalized difference vegetation index (NDVI) and radar vegetation index (RVI) were found to be highly efficient in evaluating rice production since they quantify the crop photosynthetic activity responsible for biomass formation^23^. NDVI derived from Moderate Resolution Imaging Spectroradiometer (MODIS) (AQUA/TERRA) imageries achieved a high correlation (R^2^ = 0.85) with rice production as estimated by Faisal, Rahman^24^, and R^2^ of 0.76 to 0.86 as estimated by Mosleh and Hassan^15^. SAR data captured by RADARSAT has also proved a high accuracy (97.4% and 96.6%) in estimating rice production based on back-scatter^25^.

Although several studies have discussed the use of machine learning in rice yield prediction, hybrid models that integrate two models are still poorly documented. In addition, integrating multi-data sources such as climate data, remote sensing, and agricultural statistics in rice yield estimation is poorly tested. Therefore, the present study aims to (1) Develop multiple single and hybrid machine learning models for predicting rice production across China, the world's biggest rice producer, producing 211 million tons^26^ to test multi-input scenarios (climatic variables, remote sensing, agriculture statistics and soil properties) to define the optimal combination of input variables to generate the most accurate rice production model. (2) Select the main dominant factors (climate, soil, remote sensing and sown area) that influence the rice production in each zonal scale. (3) Introduce optimal solutions for improving rice production across China. This research is critical in determining the best approach (optimal model and input variables) that could be used as a simple, rapid, and inexpensive approach for timely and reliable rice production prediction at regional scales across China. Therefore, the main contributions of the research paper are as follows.

1. This study attempts to model and predict rice production using multi-source data and hybrid machine-learning algorithms.
2. This study provides an in-depth comparative analysis of the proposed hybrid model with single machine learning models such as random forest (RF), extreme gradient boosting (XGB), conventional neural network (CNN) and long short-term memory (LSTM), and the hybrid RF-XGB and CNN-LSTM algorithms with eleven combinations (scenarios) of input variables across China.
3. This study investigates and figures out the main dominant factor for rice production across China’s main rice counties based on multi-input scenarios (climatic variables, remote sensing, agriculture statistics and soil properties).

## Materials

### Study area

**Figure 1:**
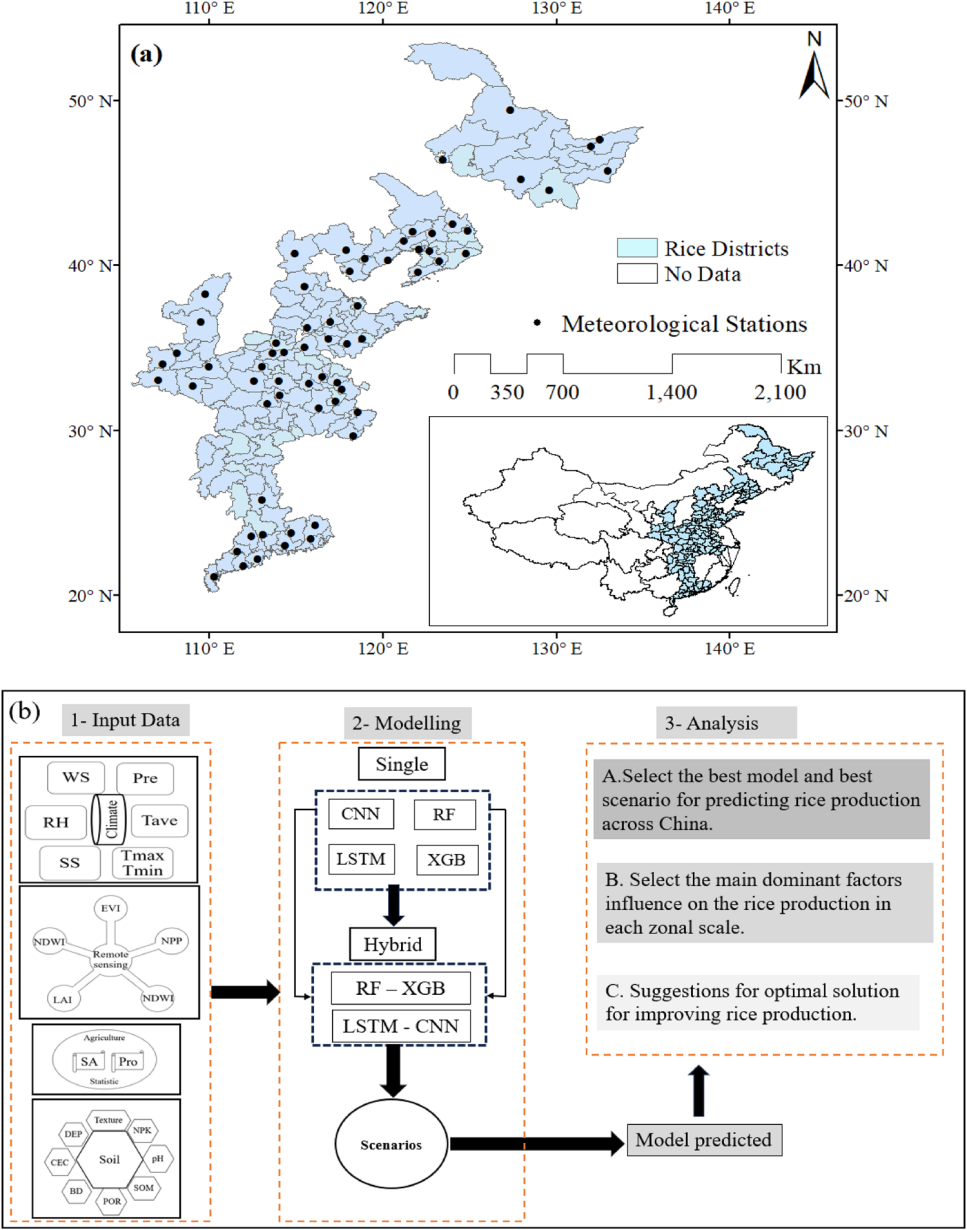
(**a**) China’s rice districts and distribution of meteorological stations, (**b**) the flowchart of methodology. The map in Fig. 1a was generated with the ArcGIS10.8 software and (**b**) was generated based on Microsoft PowerPoint.

In this study, we focused on the main cultivation areas of rice in mainland China, dominated by single-rice system (i.e. one rice harvest per year in a given field) and double-rice system (i.e. two rice harvests per year in a given field) (Fig. 1). The study area covers approximately 29 million hectares in nine provinces. This region, between 20° 10′ N ~ 53° 33′ N and 105° 54′ E ~ 135° 05′ E, is the most important food basket in China, accounting for ~ 96% of the total rice cultivation area and ~ 94% of the total rice production in China^27–29^. China, the world’s largest rice producer (about 206 million metric tons of annual production), accounts for 28% of the world’s rice production^30^. Rice occupies 41% of total grain production with only 35% of the cropland areas in China, which feeds roughly 65% of Chinese people^31^. The nine provinces are Heilongjiang, Shaanxi, Liaoning, Hainan, Anhui, Hebei, Henan, Guangdong and Shandong. The large difference in latitude leads to a pronounced variation in illumination conditions during the year: in South China, the minimum and maximum daily sunshine duration are 11 and 13 h while in North China they are 7 and 17 h, respectively. Due to its location at the eastern margin of the Eurasian continent, the climate of the eastern part of China is monsoonal with warm and humid summers and temperate, dry winters.

### Datasets

The monthly meteorological datasets over the rice districts in nine provinces across China were retrieved from the China National Meteorological Data Sharing Platform^32–35^. The data on rice production and sown area of 64 rice districts from 2000 to 2017 were extracted from the National Bureau of Statistics of China (Table 1). Moreover, for the remote sensing datasets, three vegetation indices (VIs) and two biophysical parameters (BPs) were used in the present study to estimate rice production. These five parameters are available on Google Earth Engine (GEE, https://developers.google.com/earth-engine/datasets/) with a spatial resolution of 500 m. The VIs were widely used in earlier studies as production estimators due to their relevance to vegetation health^18,36,37^. BPs were also used in wheat yield prediction^3^. Compared to VIs, the BPs are usually more reliable in estimating crop production since they more adequately reflect the state of the crops and thus could be more accurate in predicting crop yield and production. The present study used GEE to estimate the average annual value of all five parameters over the 64 rice districts in China. In addition to weather data, soil properties including soil depth, soil organic matter, pH, cation exchange capacity, porosity, bulk density, NPK and soil texture for the topsoil layer (0–30 cm) and the subsoil layer (30–100 cm) at 0.00833° (~ 1 km) were also collected and detailed in http://globalechange.bnu.edu.cn Ref.^38^.

**Table 1:** Summary of the collected datasets.

Category	Variables	Spatial resolution	Temporal resolution	Time coverage	Source
Climate data	T_max_, T_min_, Tave, Pre, RH, WS, SS)	1 km	Daily	2000–2017	China National Meteorological Data Sharing Platform (http://data.cma.cn/en)
Remote sensing data	NDVI, EVI, LAI, NDWI NPP	500 m	16-day Yearly	2000–2017	MODIS Terra Daily (https://lpdaac.usgs.gov/data/)
Production Rice (Pro) and Swon area (SA)		County	Year	2000–2017	National Bureau of Statistics of China (www.epSchinadata.com/)
Soil data	SOM, pH, DEP, POR, BD, NPK, Texture, CEC	1 km	–	–	Ref.^38^ (http://globalechange.bnu.edu.cn)

*T*_max_ maximum temperature, *T*_min_ minimum temperature, *Pre* precipitation, *RH* relative humidity, *WS* wind speed, *SS* sunshine, *NDVI* normalized difference vegetation index, EVI Enhanced vegetation index, *LAI* leaf area index, *NPP* net primary productivity and *NDWI* normalized difference water index, *DEP* soil depth, *SOM* soil organic matter, *pH POR* porosity, *BD* bulk density, *N* nitrogen, *P* phosphorus, *K* potassium, *CEC* cation exchange capacity.

## Methodology

The general methodology of the present study is shown in Fig. 1b. The study used multi-data sources, including remote sensing, climate data, agriculture statistics and soil properties data, as input variables to single and hybrid algorithms to predict the rice production. Description of the developed single and hybrid models in this work was presented as follows:

### Single models

#### Extreme gradient boosting (XGB)

The XGB algorithm suggested by Ref.^39^ is a novel improvement of the Gradient Boosting Machine based on regression trees. The algorithm is based on the idea of “boosting”, which combines all the predictions of a set of “weak” learners to develop a “strong” learner through additive training strategies, for more detailed information and the computation procedures of the XGB algorithm can be found in Ref.^39^. We applied the XGB by using the grid search method for different n estimators (number of trees) and max depth.

#### Random forest (RF)

The RF model, developed by Breiman^40^, is based on an ensemble of decision trees with controlled variance. The RF model has been widely used for regression and classification problems Such as land use/cover mapping^41^ and water quality field^42,43^. The detailed data and computation procedure of the RF model can be found in Refs.^40,44^.

#### Long short-term memory (LSTM)

LSTM is a special type of recurrent neural network (RNN)^45^ used to handle sequential data with advantages over traditional RNN. An LSTM network contains different memory blocks, which are linked through layers. Each layer includes a set of frequently connected memory pixels and three multiplicative units, namely the input, forget, and output gates^46,47^. The Adam training algorithm was used; the learning rate was set to 0. 0001 and the batch size was set to 5^48^.

#### Conventional neural network (CNN)

The convolution layers are the main difference between CNN and conventional ANN. These layers can perform automatic feature extraction, capturing features of the input data, which are key to figuring out the relationship between the inputs and output parameters. In this study, CNN with one-dimensional (1D) conventional filters (1D CNN) was used^44,49^. Detailed information about the CNN architecture and specification can be found in Ref.^33,50,51^.

### Hybrid models

#### Hybrid RF and XGB

The hybridization between the RF model and the XGB aimed to improve the performance of single models. Every single model was described in the previous sections. The use of RF-XGB reported high accuracy compared to other ML models (e.g. ANN and SVM) in agricultural applications, such as determining irrigation timing^52^ and detecting plant diseases^53^. Hence, the present study aims to test the performance of the RF-XGB hybrid model in predicting rice yield compared to single models.

#### Hybrid LSTM and CNN

LSTM and CNN were trained with the same input and hybrid to forecast results. The proposed hybrid CNN-LSTM model uses CNN layers for feature extraction from the input data with LSTM layers for sequence learning. CNN and LSTM are the most commonly used deep learning models. The present study aimed to test the efficiency of the hybrid LSTM–CNN model in rice yield forecasting. The hyper-parameters of the hybrid LSTM–CNN model, including the training algorithm, learning rate, batch size, and the number of training epochs, were set to be similar to the single CNN and LSTM models' hyper-parameters, as explained earlier.

### Input scenarios and performance evaluation

This study investigated eleven input scenarios, including various combinations of climatic, soil, agricultural and remote sensing variables. To accurately predict rice production and evaluate each variable’s contribution, the multi-data sources were divided into eleven scenarios to figure out different solutions to predict rice production based on the available data (Table 2). There are two main methods for selecting the inputs combination: based on previous studies which trained and tested multi scenarios to achieve the best combination to arrive at the optimal combination with high accuracy, performance, and less error. The second approach depends on training and testing various variable combinations as we followed in the study to select the best scenarios in the prediction of rice production. For each scenario, we tried to apply some parameters to figure out the weight and the significance of each scenario, for example, in scenario 1, we applied only the sown area as one of the main variables affecting the rice production based on the previous studies. For other scenarios such as scenarios 3, 4 and 6 to illustrate the impact on the soil, climate remote sensing parameters on the rice production in order to figure out some best management for ensure food security in China. Other scenarios are a combination of the important parameters from climate, soil, and remote sensing together. The input datasets were divided as 70% for training and 30% for testing. Performance statistics such as the root mean square error (RMSE), Nash–Sutcliffe model efficiency coefficient (NSE), the mean absolute error (MAE), and coefficient of determination (R^2^) were used to assess the performance of applied models. The performance statistics equations are defined as:

1 $$RMSE=\sqrt{\frac{1}{n}{\sum \left({P}_{i}-{O}_{i}\right)}^{2}},$$

2 $$NSE=1-{\frac{\sum \left({P}_{i}-{O}_{i}\right)}{\sum (\overline{o}-{O}_{i}{)}^{2}}}^{2},$$

3 $$MAE=\frac{1}{n}{\sum }_{i=1}^{n}\left|{O}_{i}-{P}_{i}\right|,$$

4 $${R}^{2}={\left[\frac{{\sum }_{i=1}^{n}({O}_{i}-\overline{o})({P}_{i}-\overline{P})}{\sqrt{\left({{\sum }_{i=1}^{n}({O}_{i}-{\overline{o}}_{i})}^{2}\right)\left({\sum }_{i=1}^{n}({P}_{i}-\overline{P}{)}^{2}\right)}}\right]}^{2},$$

where Oi and Pi are the actual and the predicted production, respectively, $\mathop O\limits^{ - }$ representing the average values of the actual production, and i is the number of observations.

**Table 2:** Input combinations (scenarios) for the applied models.

Scenario	Inputs
Sc1	SA
Sc2	Sunshine, Tmin, Tmax and SW
Sc3	Soil variables (pH, BD, porosity, CEC, DEP, SOM, clay, sand, TN, TP, TK)
Sc4	Climate variables (Pre, Sunshine, Tave, Tmin, Tmax, WS and RH)
Sc5	Climate variables + SA
Sc6	Remote sensing (EVI, LAI, NDVI, NDWI, NPP)
Sc7	Remote sensing + SA
Sc8	Soil variables + SA
Sc9	Pre, Sunshine, SA, NDVI, NDWI
Sc10	Pre, Sunshine, Tave, Tmin, Tmax, WS, RH, Kc, ETc, EVI, LAI, NDVI, NDWI and NPP
Sc11	Climate + Soil + Remote sensing + (Kc, ETc and SA)

### The standardized yield residuals series (SYRS)

Crop yield is affected by many variables besides climate, and shows a positive trend^54,55^. Moreover, mechanization and innovation in agriculture have increased in the last century due to the following factors^55^. To remove bias introduced by non-climate factors, the original yield timeseries were transformed to standardized yield residuals series (SYRS)^56,57^. The indicator of agricultural drought risk is given by the residuals of the detrended yield $y_{i}^{T}$ as Ref.^55^:

5 $$y_{i}^{T} = y_{i}^{0} - y_{i}^{(\tau )} ,$$

where $y_{i}^{0}$ is the observed crop yield and $y_{i}^{(\tau )}$ is the value of the fitted quadratic polynomial regression model. The SYRS is computed as:

6 $$SYRS = \frac{{y_{i}^{(T)} - \mu }}{\sigma },$$

where μ is the mean of the yield residuals and σ is the standard deviation of the yield residuals^55^.

The percentage of annual yield loss was based on Eq. (7). SPEI-3 and SPEI-6 were analyzed to assess the effect of drought severity and to evaluate the vegetation response to drought^58^. To assess the impacts of drought on crop yields, changes in the percentage of annual yield loss (Y_L_%) was estimated as:

7 $$Y_{L} = \frac{{Y_{i}^{0} - Y_{i}^{(\tau )} }}{{Y_{i}^{(\tau )} }} \times 100,$$

## Results

### Model performance

#### Performance of the single and hybrid models

To compare the accuracy of the single and the hybrid models, this study tested the performance of the four single models (RF, XGB, CNN, and LSTM) against the two hybrid models (RF-XGB and CNN-LSTM). Overall, hybrid models have performed better in estimating rice production than single models as the average of all input scenarios (Table 3). It is also notable that the use of the sowing area alone achieved a relatively high-performance estimation with an average R^2^ of 0.825, NSE = 0.823, and RMSE = 35.592 × 10^4^ ton, among all ML methods. Without SA, the integration of both climatic and remote sensing achieved a moderate performance (Sc10, R^2^ **=** 0.533 (Table 3). The highest R^2^ (0.8593) and NSE (0.8556), and the lowest RMSE (26.6903 × 10^4^ ton) were achieved by the hybrid RF-XGB model, followed by LSTM-CNN. In contrast, the lowest model performance was the LSTM model by 0.6786, 0.6693 and 43.9143 × 10^4^ ton for R^2^, NSE and RMSE respectively.

**Table 3:** The performance evaluation of applied models in rice production.

Models	R^2^	NSE	RMSE (× 10^4^ ton)
RF	0.767	0.762	38.359
XGB	0.760	0.756	38.374
**RF-XGB**	**0.859**	**0.856**	**26.690**
LSTM	0.757	0.755	39.200
CNN	0.679	0.669	43.914
**LSTM-CNN**	**0.851**	**0.850**	**29.052**
Scenario			
Sc1	0.825	0.823	35.59
Sc2	0.895	0.900	26.92
**Sc3**	**0.883**	**0.872**	**28.68**
Sc4	0.498	0.489	58.30
Sc5	0.899	0.898	26.31
Sc6	0.362	0.340	68.66
Sc7	0.894	0.892	27.32
**Sc8**	**0.950**	**0.950**	**18.69**
Sc9	0.881	0.879	28.69
Sc10	0.533	0.529	56.80
**Sc11**	**0.948**	**0.948**	**19.30**

The values in the table were estimated as averages for the applied models and input scenarios. Significant values are in bold.

#### Optimum input scenario for rice production

According to the performance’s results of the applied models, The tested models showed variant performance among the various input scenarios. On average, the best scenario was observed in scenario 8 (soil variables and sown area) and 11 (All variables) as inputs to the prediction models (Table 3). In both scenarios 8 and 11, the R^2^ and NSE were 0.95 and the RMSE was 19.69 × 10^4^ ton and 19.3 × 10^4^ ton for respectively. On the other hand, the use of remote sensing indices alone achieved the lowest performed scenario (Sc6) for rice production estimation (R^2^ = 0.362, NSE = 0.340, RMSE = 68.659 × 10^4^ ton), while the use of sown area with remote sensing (scenario 7), the performance of the models was enhanced significantly (R^2^ = 0.899, NSE = 0.898, RMSE = 27.32 × 10^4^ ton).

**Figure 2:**
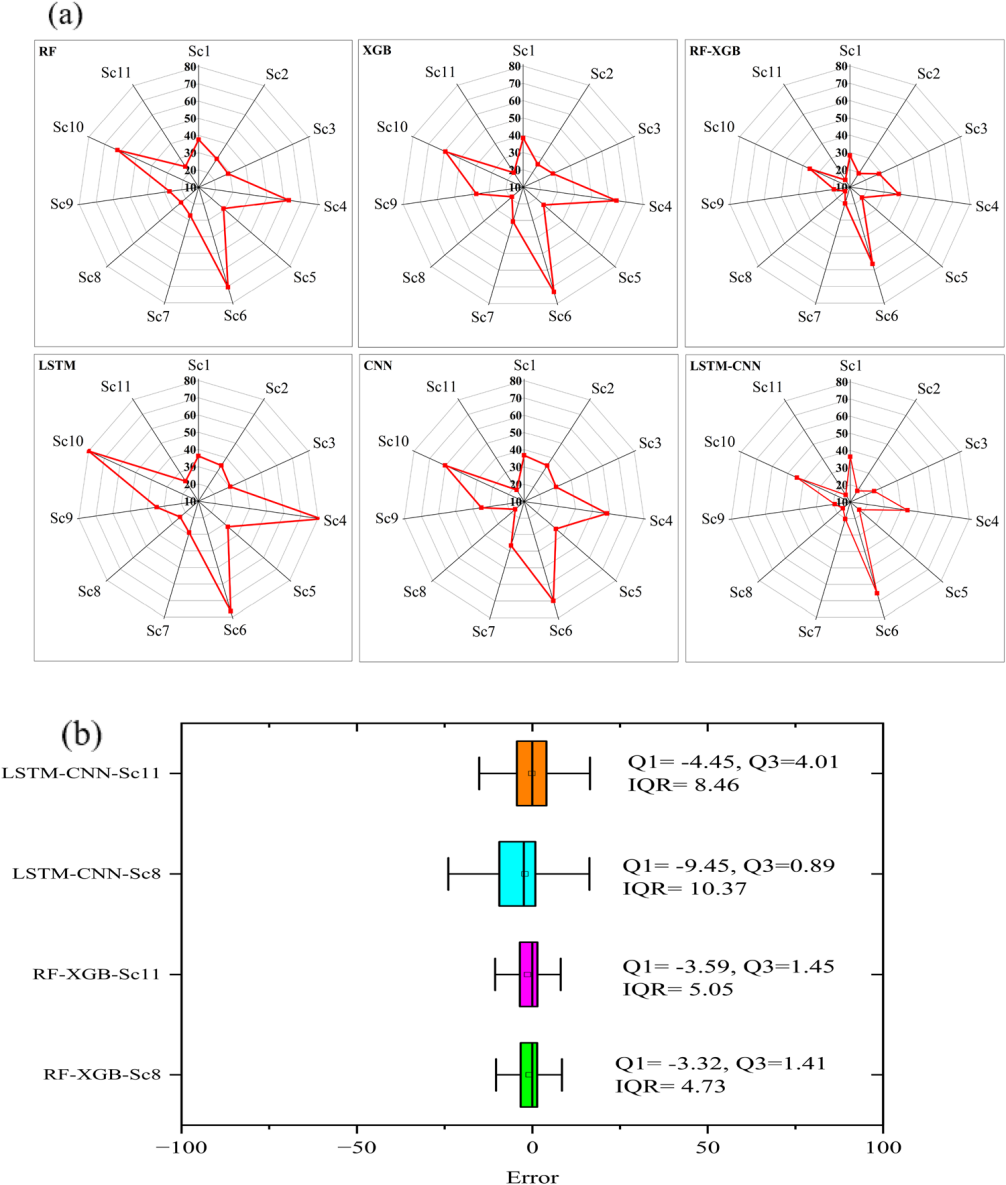
Radar chart for the RMSE of the applied models (**a**), the boxplot of the RF-XGB and LSTM-CNN models (Sc: scenario), (**b**) The boxplot of error distribution of the developed RF-XGB and LSTM-CNN models at scenarios 8 and 11. The figures were generated with the Origin 2023b software.

To investigate the performance of each model (single and hybrid models) under the eleven scenarios, R^2^, NSE and MAE indices were calculated for the different scenarios in the applied models (Table 4). The lowest single model was LSTM in scenarios 10 and 4 by MAE (51.38 × 10^4^ and 50.35 × 10^4^ ton) respectively. Meanwhile, the highest performance model was RF-XGB in scenarios 8 (soil variables and SA) and 5 (climate variables and SA) by MAE (5.85 × 10^4^ and 7.70 × 10^4^ ton), respectively. In contrast, the highest R^2^ values were recorded in scenarios 8 and 11 by 0.97 for RF-XGB and LSTM-CNN and the lowest R^2^ values were in scenario 4 (climate variables) in the LSTM model followed by scenario 10 by 0.11 and 0.13. Moreover, the NSE index indicates that the highest model was RF-XGB and LSTM-CNN by 0.97 for both models in scenarios 8 and 11. The lowest NSE values were 0.27 and 0.32 in scenario 6 (remote sensing) with XGB and RF models respectively. The scenario 3 (soil variables), the NSE was higher 0.82 for all models, while the NSE was enhanced in scenario 11 to be higher than 0.92 for all models. The highest NSE values were recorded in scenarios 8 and 11 by 0.97 for RF-XGB and LSTM-CNN. In contrast, the Radar chart shows the RMSE for the applied models in the different scenarios (Fig. 2a), the lowest single model was LSTM in scenario 4 (climate variables) by RMSE (81.85 × 10^4^ ton), followed by the XGB in scenario 6 (remote sensing) by RMSE (73.13 × 10^4^ ton) (Fig. 2a). However, the performance accuracy in these two scenarios was enhanced when applying the hybrid model, for example, scenarios 4 and 5 with the RF-XGB model achieved RMSE 38.45 × 10^4^ ton and 6.45 × 10^4^ ton by respectively, which enhanced by model by RMSE 13.65 × 10^4^ ton, followed by scenario 11 (All variables) with LSTM-CNN and RF-XGB models by RMSE 14.90 × 10^4^ ton. Based on the results, it is clear that the hybrid models performed better in rice production estimation than single models. On one hand, the lowest performance in all scenarios on the hybrid models was in scenarios 6 (remote sensing) and 4 (climate) respectively. On the other hand, the highest performance in all scenarios was in scenarios 8 and 11 respectively.

**Table 4:** The performance evaluation of applied models in rice production.

	Sc1	Sc2	Sc3	Sc4	Sc5	Sc6	Sc7	Sc8	Sc9	Sc10	Sc11
R^2^											
RF	0.81	0.88	0.89	0.47	0.89	0.35	0.90	0.93	0.90	0.50	0.92
XGB	0.80	0.91	0.89	0.44	0.91	0.29	0.88	0.95	0.81	0.52	0.95
RF-XGB	0.87	0.94	0.87	0.69	0.94	0.52	0.94	0.97	0.94	0.80	0.97
LSTM	0.82	0.84	0.88	0.54	0.84	0.34	0.82	0.96	0.83	0.51	0.96
CNN	0.82	0.84	0.88	0.11	0.86	0.25	0.89	0.92	0.84	0.13	0.92
LSTM-CNN	0.82	0.96	0.89	0.74	0.96	0.42	0.95	0.97	0.95	0.74	0.97
NSE											
RF	0.81	0.88	0.89	0.46	0.88	0.32	0.90	0.93	0.90	0.48	0.92
XGB	0.80	0.91	0.89	0.43	0.91	0.27	0.87	0.95	0.81	0.52	0.95
RF-XGB	0.87	0.94	0.87	0.68	0.94	0.49	0.94	0.97	0.94	0.80	0.97
LSTM	0.82	0.84	0.87	0.53	0.84	0.33	0.82	0.96	0.83	0.51	0.96
CNN	0.82	0.88	0.82	0.09	0.86	0.20	0.89	0.92	0.84	0.13	0.92
LSTM-CNN	0.82	0.96	0.89	0.74	0.96	0.42	0.94	0.97	0.95	0.73	0.97
MAE											
RF	15.3	11.4	14.3	35.1	11.7	39.7	11.5	9.4	11.8	33.1	9.8
XGB	16.3	11.1	14.2	36.9	12.8	43.8	14.1	7.9	16.8	32.6	10.7
RF-XGB	11.6	7.4	13.8	19.1	7.7	30.9	8.2	5.9	8.5	17.5	6.8
LSTM	14.9	15.4	16.8	37.9	15.0	41.1	15.1	8.4	15.9	37.9	9.8
CNN	14.7	15.9	16.1	50.4	14.9	41.7	13.2	9.8	14.0	51.4	12.3
LSTM-CNN	15.7	10.1	12.6	26.8	9.3	38.5	9.9	9.4	10.3	23.8	8.3

Therefore, to select the best hybrid models and scenario, the box plot was developed for scenarios 8 and 11 in RF-XGB and LSTM-CNN to compare the models based on the residuals (estimation error). Positive and negative estimation errors show under- and over-estimations, respectively. The RF-XGB model in scenarios 8 and 11 appears to be the best model having the lowest error by 53% and 23% in comparison with applying LSTM and XGB models, respectively in comparison with the others. On the other hand, the lowest scenario was scenario 8 (soil + SA) with RF-XGB The RF-XGB model in scenarios 8 and 11 appears to be the best model having the lowest error in comparison with the others. For scenario 8, it has a lower quartile (Q1) value of − 3.32 and for the LSTM-CNN (Q1 =  − 9.47), also, for scenario 11, the Q1 was − 3.59 and in the LSTM-CNN (Q1 =  − 4.45). Moreover, the smaller interquartile range (IQR = Q3-Q1) by the RF-XGB model compared with the LSTM-CNN model clearly shows that its distribution of error is much better than the LSTM-CNN model (Fig. 2b), it was 1.41 and 1.45 for scenario 8 and 11 respectively, however, it was 10.37 and 8.46 for LSTM-CNN model. Therefore, the RF-XGB model shows a clear superiority in scenarios 8 and 11.

### Importance of predictor variables in rice production estimation

**Figure 3:**
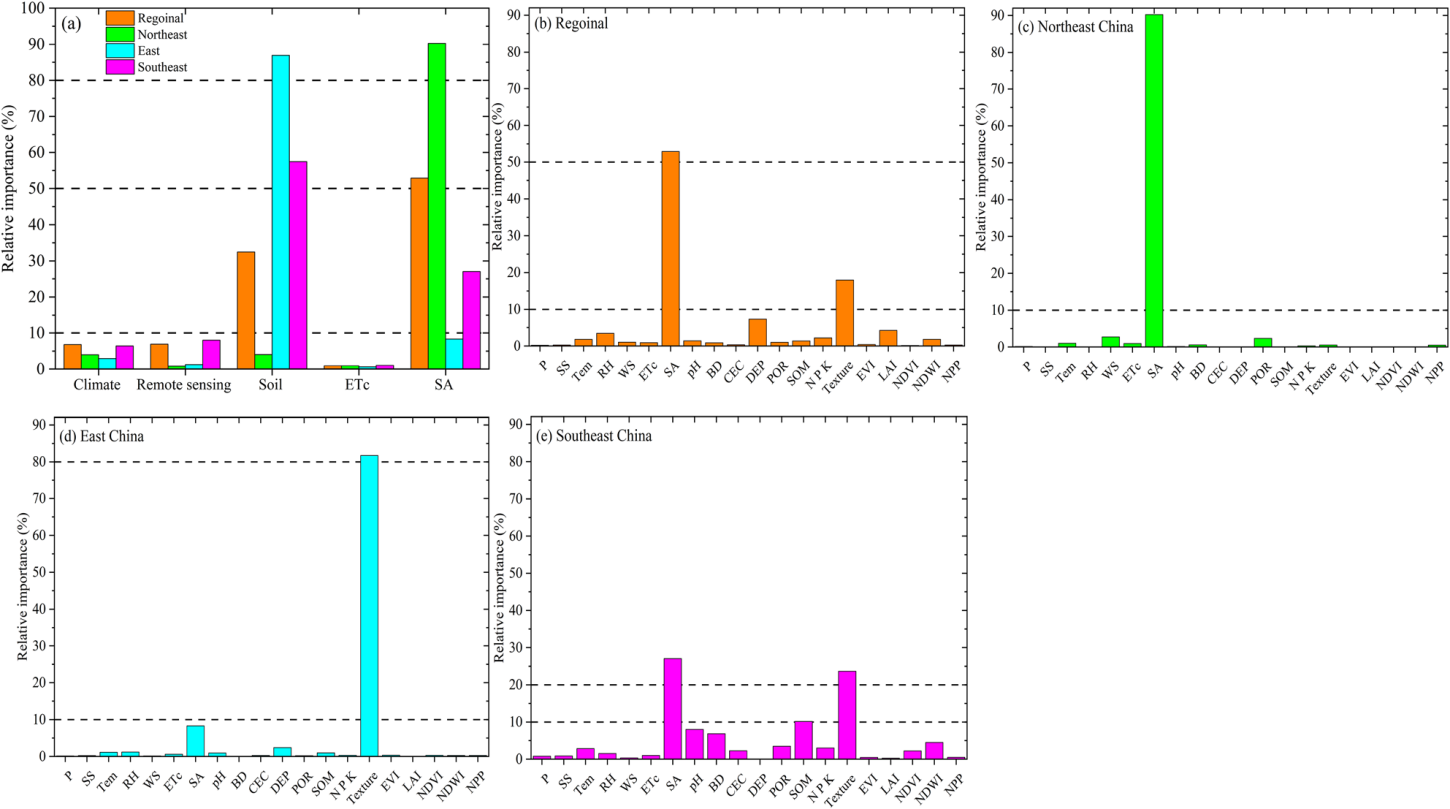
Relative importance ranking of the features in rice production estimation for the regional and zonal scale. The figures were generated with the Origin 2023b software.

Based on the results obtained from the single RF and XGB models, it is the superiority of the XGB model in comparison with the RF model, thus, the XGB model was applied to analyse the joint contributions of subsets of features while maintaining a fast convergence during iterations. The predictor variables in the XGB model were used to investigate the importance of these predictor variables. The importance ranking of predictor variables for the regional and zonal scale showed that it had different effects or importance on rice production estimation (Fig. 3). For the regional scale, the most important feature in the rice estimation was sown area by 53%, followed by soil properties (32%), and climate (7%) (Fig. 3a). The importance of the sown area decreased to by 8% and 27% respectively. On the other hand, the sown area was very significantly important in the rice production estimation in northeast China and southeast China by 90% and 27% respectively. Therefore, to separately analyze the factors of climate, soil and remote sensing, Fig. 3c–e were developed. For example, the importance of the soil texture contributed 18% of the total contribution of the soil properties (32%) for rice production estimation across China. While the percentage of the contribution increased significantly by 82% in East China from the total contribution of the soil properties (87%), however, the contribution of texture was 24% in South China. In contrast, the contribution of climate change was low in all zones, the relative humidity contributed 3.5% of the total contribution of the climate on the regional scale, however, in southeast China, the temperature contributed almost half of the total contribution of the climate (2.95%) (Fig. 3e), evapotranspiration was at the bottom of the importance ranking due to the low importance of the climate factors. Meanwhile, for the zonal scale, in northeast China, the importance of sown area increased to be the main dominant factor for rice production estimation reaching 90% followed by soil properties by 4% (Fig. 3b). On the other hand, the soil properties were the main dominant factor impacting on rice production in east and southeast China by 87% and 57% respectively.

### Solution for improving rice production

**Figure 4:**
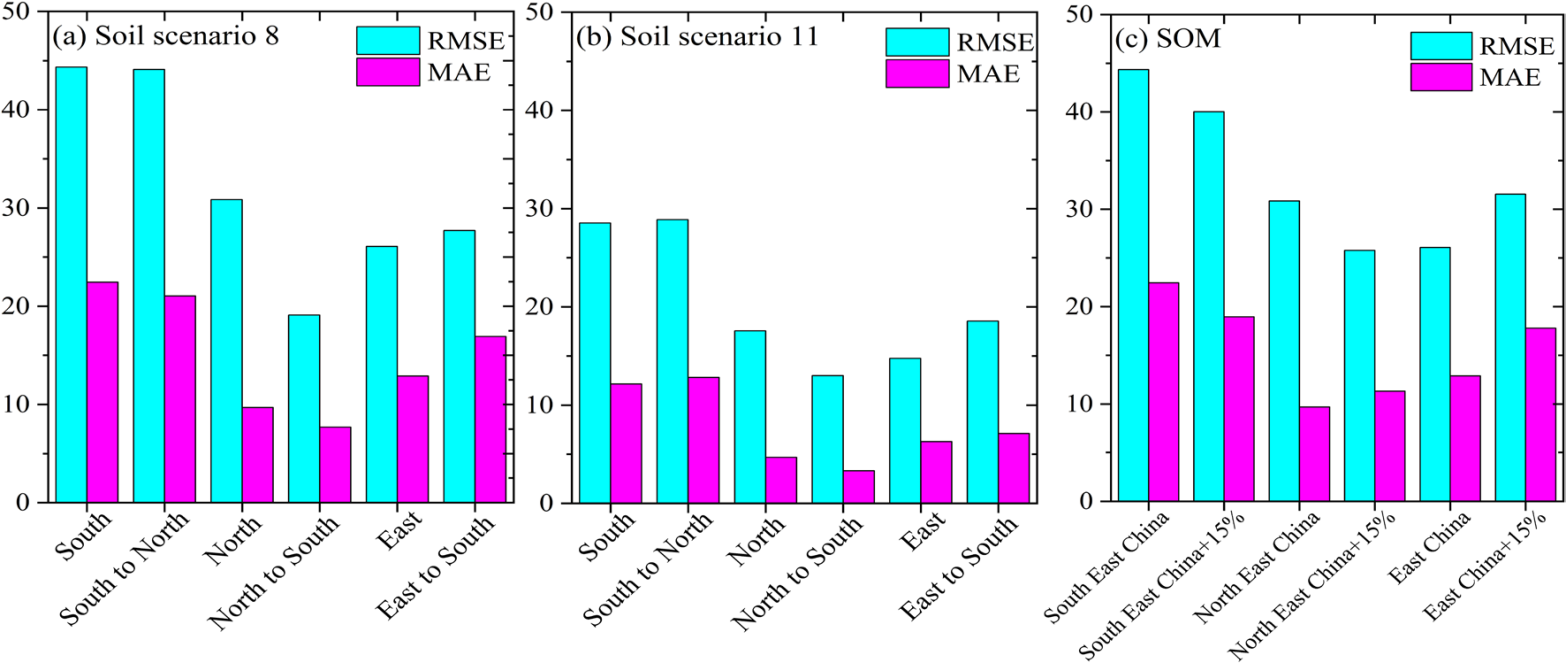
Changing the soil properties (**a**,**b**) and increasing SOM by 15% (**c**) in each zone. The figures were generated with the Origin 2023b software.

To improve the rice production in each zone, we exchanged and alignment of the soil properties from northeast China to southeast China and from east to southeast China. Figure 4a shows the variation of changing the soil properties in scenario 8, the RMSE decreased by 38% in northeast China when changed the soil properties to southeast China. In contrast, when the soil properties in southeast, China changed to the northeast, China, the RMSE did not significantly decrease (0.6%). In the same manner, the MAE was significantly decreased when changed the soil properties of northeast China to southeast China by 20%. Scenario 11 was consistent with scenario 8, the RMSE significantly decreased when the soil properties of northeast China to southeast China changed by 26% (Fig. 4b). On the other hand, when simulating the soil properties in east China by using the soil properties from southeast China, the performance of the model decreased, for example, the RMSE and MAE increased by 6% and 31% respectively. In contrast, one of the major suggested solutions is to increase the soil organic matter to enhance rice production. Therefore, we simulated the effect of increasing the soil organic matter by 15% on rice production (scenario 8). Figure 4c shows the performance of the hybrid RF-XGB model was enhanced significantly when increasing the SOM in northeast and southeast China by 15%, the RMSE declined by 16% and 10% respectively in comparison with the current SOM. However, increasing the SOM in East China resulted in a negative effect on the rice production estimation, the RMSE increased by 21%.

**Figure 5:**
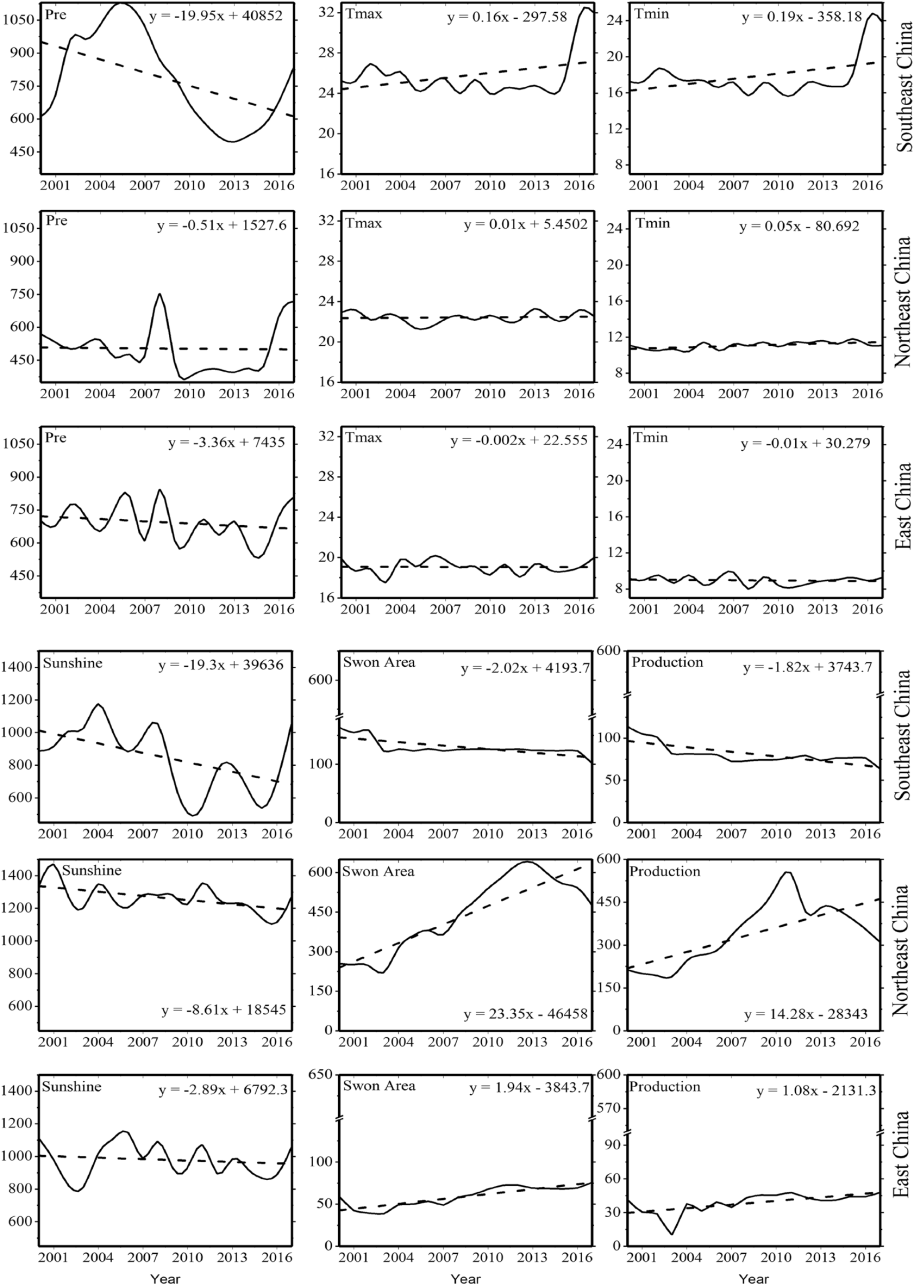
Time series of precipitation, maximum and minimum temperature), sunshine, sown area and production across zones. The figures were generated with the Origin 2023b software.

On the other hand, as shown in Fig. 5, the decreasing trend of precipitation and increasing temperature in southeast China impacted negatively rice production. The maximum and minimum temperatures increased by 0.16 and 0.19 °C/ year, while precipitation decreased by 20 mm/year which resulted in decreasing the rice production by 2.23% as average in southeast China. In contrast, the production increase in northeast China may be the reason back to the non-significant decreasing and increasing trend in precipitation and temperature (maximum and minimum) and improving irrigation that will positively affect rice production even during dry years^59,60^. Therefore, the SPEI drought index was analyzed to investigate the drought situation during the period and how it is related to the production anomaly.

**Figure 6:**
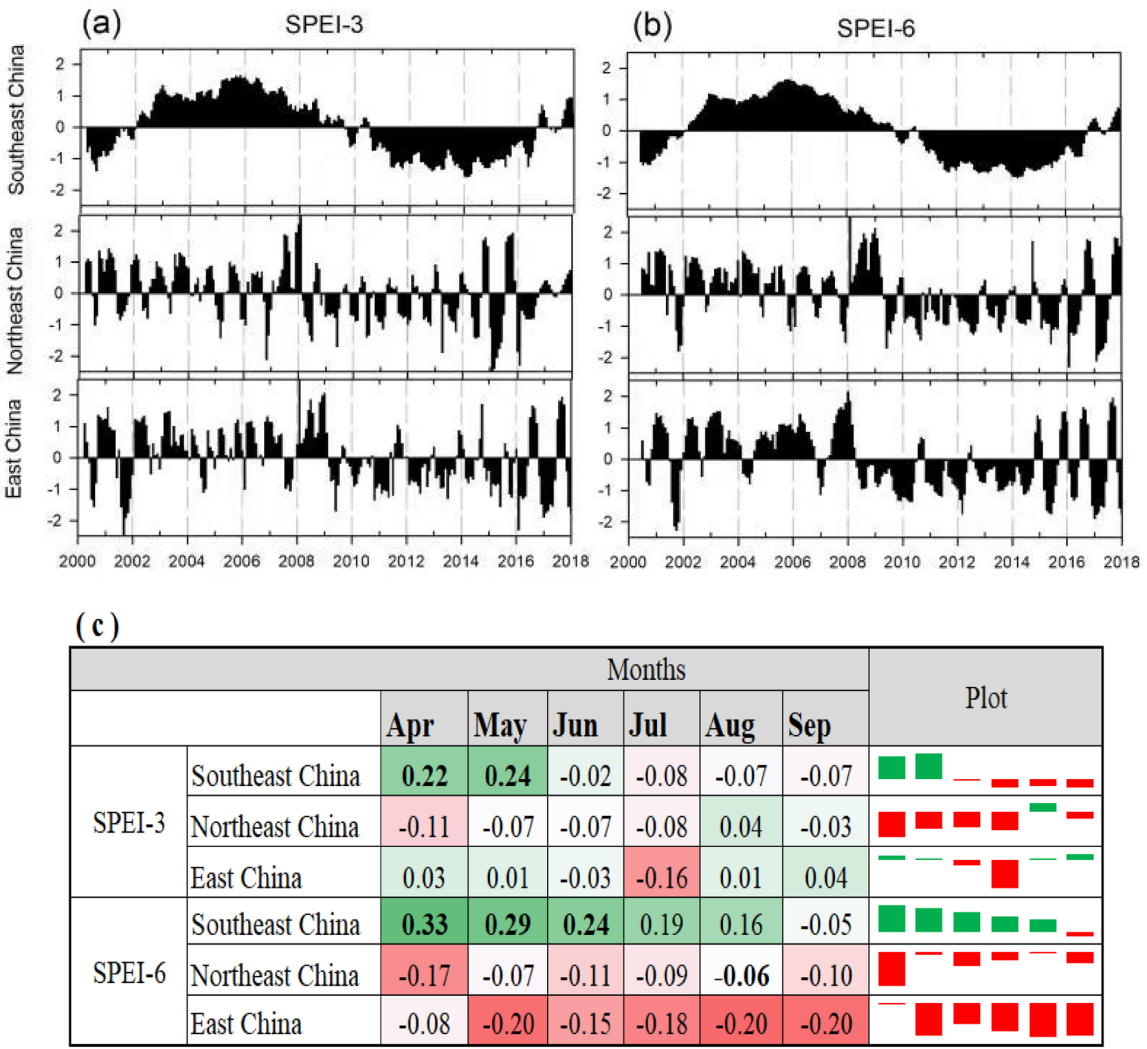
The temporal evolution of SPEI-3 and SPEI-6 (**a** and **b**), the Pearson correlation coefficient (r) of the linear regression between the SPEIs at 3- and 6-month timescale and the SYRS of rice yield in the three zones (**c**) and yield losses across the three regions (**d**). The figures were generated with the Sigma plot software.

The temporal evolution of SPEI series at 3- and 6-month timescales fluctuated during the study period (Fig. 6a and b). In Northeast China, during the period from 2009 to 2012, the drought (SPEI-3) was classified as extreme drought, especially in 2009, it was during the months (May, June and July) of the rice season. However, in East China, during the period from 2009 to 2013, the drought can be classified as severe drought. Meanwhile, in southeast China, during the period from 2011 to 2015, the drought can be classified as severe drought, however, the extreme drought was found only in 2011 for June and September months.

**Figure 7:**
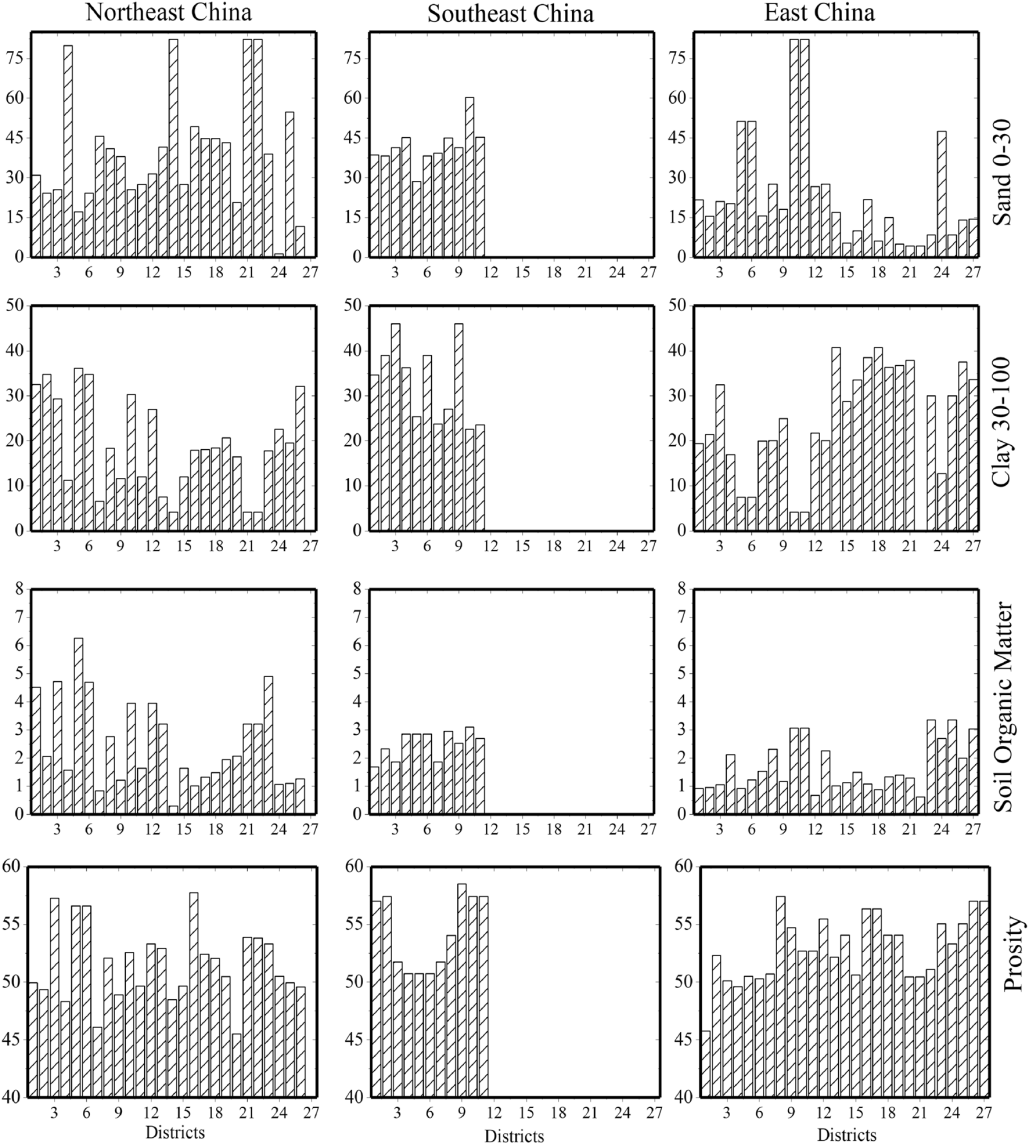
Variations of sand (0–30), clay (30–100), soil organic matter and porosity in each district. The figures were generated with the Origin 2023b software.

On the other hand, in the period from 2002 to 2008, there was no drought event happened during this period. In contrast, Fig. 6c shows the correlation analysis between the SPEI-3/6 and SYRS of rice yield across the three zones. The correlation coefficient between SYRS of rice in southeast China and SPEI in April and May (initial stage) is the highest among all months, revealing that rice yield is more prone to drought in the initial stage. Meanwhile, in northeast and east China, the rice yield is less correlated with drought than in southeast China, which may be the reason back to the improving irrigation will positively affect rice yield even during dry years. It is observed that the degree of yield losses varies during the study period across the three regions due to drought/wet impact on the various crop stages. In East China, 2003 ranked as the year with the highest failure of rice, the yield losses reached to 60%. In contrast, in southeast China, the highest losses occurred in 2001, 2002 and 2003 by 20%, 27% and 18%, with average losses during the whole study period by 2.23% (Fig. 6d). Besides the climate variables, soil properties play a vital role in improving rice production. The results from this study indicated that the clay (30–100 cm) was positively correlated with the rice production in the three zones, especially in northeast China (Fig. 7). It was the same in the sand (0–30 cm) in southeast China, however, it was negatively in the northeast and east China.

## Discussion

### Hybrid method importance in rice yield estimation

The results from this study documented that the hybrid models RF-XGB and LSTM-CNN models are more flexible and robust with noisy data than single models, significantly enhancing their prediction accuracy. Previous researches have documented that both climate variables and remote sensing data could exert non-linear and complicated effects on production variations^61^, which however could be less captured by the single methods. For example, the RMSE was reduced by more than 30% when applying hybrid the RF-XGB and LSTM-CNN models compared to the single models, which agrees with the findings of Chiu, Wen^61^. The underlying reason may be that using a single machine learning aggressor may result in over-fitting and difficulty with generalization. This is because the regressor may become too complex and fit the noise in the training data, rather than the underlying patterns^62–64^. Further, Huang et al.^65^ developed, trained, and tested a back-propagation neural network (BP-ANN) model for fiber-reinforced polymer (FRP) reinforced concrete at high temperatures using 151 sets of FRP-reinforced concrete pullout test data at different temperatures reported in the literature. The results showed that the BP-ANN model exhibited greater generality than existing mathematical models. Furthermore, Wang et al.^63^ combined ANN with genetic algorithm (GA) or particle swarm optimization (PSO) for model training and testing. The findings indicated that the accuracy of the developed hybrid machine learning model in predicting bond strength in CES structures exceeded that of conventional ANN models and existing empirical equations. In addition, both DL and ML models are black boxes. It is difficult to produce testable hypotheses that could potentially provide biological insights because of their complex model structure. In contrast, in comparison with traditional production estimation methods (i.e. crop models simulation and statistical regression), the ML and DL methods provide new opportunities for yield predictions^27^. However, combining crop models and DL/ML models for yield estimation, forecasts, and disaster monitoring in large regions is recommended. This might encourage running the models of rice production estimation at the local scale to consider the variation among rice districts in their agro-environmental conditions and the relative correlation of various factors with rice production.

### Analysis of driving mechanisms on rice production

The global warming phenomenon has undoubtedly brought unprecedented challenges to rice production, vital for food security in southeast Asian countries and China. The excessively high temperature will increase the risk of heat stress, which will not only make others challenging to crack but contribute to the reduction of pollen, thus affecting the normal process of pollination and fertilization. Meanwhile, excessive heat will inhibit rice from synthetic organic matter and accumulate dry matter, leading to reduced seed setting rate, grain mass, and seed weight^66–68^. A reduction in rainfall will decrease the stomatal conductance and inter-cellular CO_2_ flux, which will slow down the transpiration rate and restrict photosynthesis^69^. As a result, the uptake of nutrients will be reduced, and respiration consumption will increase oppositely. Therefore, the increase of precipitation in a moderate range can promote rice yield. Our findings agree with the findings of Liu et al.^70^, who reported that the individual contribution of climate change, soil improvement to rice yield differed with respected factors. Compared with the 1980s, the yield in the 2000s decreased by 19.5% from climate change, while the yield increased by 12.7% due to soil improvement. In contrast, the increase in rice production in northeast China may be the reason back to the non-significant decreasing and increasing trend in precipitation and temperature (maximum and minimum) and adequate irrigation and adjusting sowing dates that will positively affect rice production even during dry years^59,71–73^ and also, the appropriate application of chemical fertilizers, providing ample nutrients to the growth of rice^74^. As shown in Fig. 6a and b), in southeast China, during the period from 2011 to 2015, the drought can be classified as severe drought, however, the extreme drought was found only in 2011 for June and September months. Furthermore, the correlation coefficient between SYRS of rice in southeast China and SPEI in April and May (initial stage) is the highest among all months, revealing that rice yield is more prone to drought in the initial stage. Meanwhile, in northeast and east China, the rice yield is less correlated with drought than in southeast China, which may be the reason back to the improving irrigation will positively affect rice yield even during dry years^72,73^. Furthermore, the role of climatic variables in rice yield variation was not significant in some regions in China, these results are supported by some previous studies^75,76^. The underlying reasons may be that sown area and soil properties represent comprehensive features or information of a county or a field over a long time, while climate factors represent a part of the information related to crop production for a specific period. In contrast, high production can be characterized by healthy soils, well water conditions, farmer's experiences, agricultural practices such as applying mulches, well-equipped irrigation facilities, fertilizers and suitable climate conditions^75^. All these features can be comprehensively represented by spatial location. Furthermore, climatic variables derived from meteorological data were better in rice production estimation than vegetation parameters derived from remote sensing data. This agrees with earlier studies that the fluctuation in precipitation and temperature proved a strong correlation with rice production^21,22^. Although remote sensing vegetation indices (VIs) performed less than climatic variables in rice production estimation at the regional scale, VIs were more important than the climate in some rice districts. The explanation may be that the satellite indices can reflect the effects not only of abiotic factors but also biotic factors (e.g. plant disease, irrigation, and fertilization)^77,78^, which agree with the conclusion of Cao, Zhang^27^. Moreover, we speculate that monthly EVI and weather data cannot accurately reflect crop growth and development. The EVI at the 8-day or 16-day period might better incorporate crop growth and weather information^75^. Moreover, a subset of climatic variables in scenario 2 (Sunshine, T_min_, T_max_, and sown area) achieved comparable rice production estimation results to using full climatic variables as in scenario 5. The reason may be due to the highly significant between the sown area and rice production as shown in Fig. 7 in the three zones. In contrast, soil health is one of the major factors affecting rice production^79^. Increasing the clay content could improve soil fertility^80^. A higher biomass was recorded in rice grown in high clay soil than in rice grown in low clay soil^80,81^. Southern China accounts for 88% of national rice production^82^. Continuous flooding irrigation is practiced by Chinese farmers in lowland rice, threatening rice production^83^. Moreover, in regions of southern China, clay-textured soils offer the highest potassium-supplying potential^84^. The results from this study indicated that the clay (30–100 cm) was positively correlated with the rice production in the three zones, especially in northeast China, however, it was negative in northeast and east China. The main reason is that soil texture affects plant growth and nutrient uptake because it alters the availability of water in the soil. When the soil has high clay contents, often with a large proportion of 2:1 clay, it is classified as Vertisol^79^. In flooded rice soil, soil swelling is dominant because clay absorbs water, then the soil is allowed to dry out before irrigation is applied again^85^; as such, cracks are dominant in paddy soils^86^ due to the removal of water from within and between clay micro structures.

## Conclusion

In this study, the key issue was finding the best approach to predict rice production across China’s main rice counties by testing multiple single and hybrid models and input scenarios at various study scales. Based on the results, the main findings of the present study can be summarized as follows;

- Hybrid models performed better than single models in rice production estimation which significantly improves the prediction accuracy.
- For the zonal scale, the soil properties were the most dominant factors in rice production, it was 87 and 53% in east and southeast China respectively.
- The increase in temperature and decrease in precipitation restrain rice production by decreasing rice production by 2.2% as average in southeast China.
- At the regional scale, climatic variables showed a strong relationship with rice production than vegetation parameters. However, remote sensing outperformed climatic factors in some local districts. The paper's innovation lies in its holistic approach to predicting rice production using multi-source data and hybrid machine learning algorithms, offering high-resolution insights into a critical aspect of China's agriculture. Furthermore, one of the main innovative points of this study was to investigate the dominant factor for rice production across China’s main rice counties. In contrast, future research will focus on predicting rice production using agronomic datasets (crop phenology, growing degree days, full grain, panic number, and plant height) as well as management datasets in addition to the existing datasets.

## Data Availability

The datasets used and/or analyzed during the current study are available from the corresponding author on reasonable request.

## References

[1] Balakrishnan N, Muthukumarasamy G (2016). Crop production-ensemble machine learning model for prediction. Int. J. Comput. Sci. Softw. Eng..

[2] Mekonnen MM, Hoekstra AY (2010). A global and high-resolution assessment of the green, blue and grey water footprint of wheat. Hydrol. Earth Syst. Sci..

[3] Huang J, Xu C, Ridoutt BG, Chen F (2015). Reducing agricultural water footprints at the farm scale: A case study in the Beijing region. Water.

[4] Fan J, Jintrawet A, Sangchyoswat C (2020). The relationships between extreme precipitation and rice and maize yields using machine learning in Sichuan Province, China. Curr. Appl. Sci. Technol..

[5] Gillani SA (2019). Appraisal of urban heat island over Gujranwala and its environmental impact assessment using satellite imagery (1995–2016). Int. J. Innov. Sci. Technol..

[6] Lee S-H, Bae J-Y (2019). Predicting crop production for agricultural consultation service. J. Inf. Commun. Converg. Eng..

[7] Adnan RM, Mostafa RR, Elbeltagi A, Yaseen ZM, Shahid S, Kisi O (2022). Development of new machine learning model for streamflow prediction: Case studies in Pakistan. Stoch. Environ. Res. Risk Assess..

[8] Kouadri S, Pande CB, Panneerselvam B, Moharir KN, Elbeltagi A (2022). Prediction of irrigation groundwater quality parameters using ANN, LSTM, and MLR models. Environ. Sci. Pollut. Res..

[9] Mohammed S (2022). A comparative analysis of data mining techniques for agricultural and hydrological drought prediction in the eastern Mediterranean. Comput. Electron. Agric..

[10] Sakaa B (2022). Water quality index modeling using random forest and improved SMO algorithm for support vector machine in Saf-Saf river basin. Environ. Sci. Pollut. Res..

[11] Singh VK (2022). Novel genetic algorithm (GA) based hybrid machine learning-pedotransfer function (ML-PTF) for prediction of spatial pattern of saturated hydraulic conductivity. Eng. Appl. Comput. Fluid Mech..

[12] Carlson KM (2017). Greenhouse gas emissions intensity of global croplands. Nat. Clim. Change.

[13] Naresh R (2017). Water footprint of rice from both production and consumption perspective assessment using remote sensing under subtropical India: A review. Int. J. Chem. Stud..

[14] Zheng J (2020). Assessment of climate change impact on the water footprint in rice production: Historical simulation and future projections at two representative rice cropping sites of China. Sci. Total Environ..

[15] Mosleh MK, Hassan QK (2014). Development of a remote sensing-based “Boro” rice mapping system. Remote Sens..

[16] Jabjone S, Jiamrum C (2013). Artificial neural networks for predicting the rice yield in Phimai District of Thailand. Int. J. Electr. Energy.

[17] Marndi A, Ramesh K, Patra G (2021). Crop production estimation using deep learning technique. Curr. Sci..

[18] Sultana A, Khanam M (2020). Forecasting rice production of Bangladesh using ARIMA and artificial neural network models. Dhaka Univ. J. Sci..

[19] Koide N, Robertson AW, Ines AV, Qian J-H, Dewitt DG, Lucero A (2013). Prediction of rice production in the Philippines using seasonal climate forecasts. J. Appl. Meteorol. Climatol..

[20] Roberts MG, Dawe D, Falcon WP, Naylor RL (2009). El Niño-Southern oscillation impacts on rice production in Luzon, the Philippines. J. Appl. Meteorol. Climatol..

[21] Jianping Z (2005). Effect of climate change on the growth and yields of double-harvest rice in the Southern China. Adv. Clim. Change Res..

[22] Li W-J (2014). Climate change impact and its contribution share to paddy rice production in Jiangxi, China. J. Integr. Agric..

[23] Prasad A (2007). Use of vegetation index and meteorological parameters for the prediction of crop yield in India. Int. J. Remote Sens..

[24] Faisal BR (2019). Relationship between boro rice production and MODIS derived NDVI for rice production forecasting: A case study on Bangladesh. Dhaka Univ. J. Earth Environ. Sci..

[25] Chen C (2011). Rice area mapping, yield, and production forecast for the province of Nueva Ecija using RADARSAT imagery. Can. J. Remote Sens..

[26] Raza SMH (2018). Delineation of potential sites for rice cultivation through multi-criteria evaluation (MCE) using remote sensing and GIS. Int. J. Plant Prod..

[27] Cao J (2021). Integrating multi-source data for rice yield prediction across China using machine learning and deep learning approaches. Agric. For. Meteorol..

[28] Sun W, Huang Y (2011). Global warming over the period 1961–2008 did not increase high-temperature stress but did reduce low-temperature stress in irrigated rice across China. Agric. For. Meteorol..

[29] Zhang Z (2014). Global warming over 1960–2009 did increase heat stress and reduce cold stress in the major rice-planting areas across China. Eur. J. Agron..

[30] Deng N (2019). Closing yield gaps for rice self-sufficiency in China. Nat. Commun..

[31] Peng S, Tang Q, Zou Y (2009). Current status and challenges of rice production in China. Plant Prod. Sci..

[32] Mokhtar A (2022). Assessment of the effects of spatiotemporal characteristics of drought on crop yields in southwest China. Int. J. Climatol..

[33] Mokhtar A (2021). Estimation of SPEI meteorological drought using machine learning algorithms. IEEE Access.

[34] Mokhtar A (2021). Ecosystem water use efficiency response to drought over Southwest China. Ecohydrology.

[35] Mokhtar A (2021). Estimation of the rice water footprint based on machine learning algorithms. Comput. Electron. Agric..

[36] Han H, Armaghani DJ, Tarinejad R, Zhou J, Tahir M (2020). Random forest and bayesian network techniques for probabilistic prediction of flyrock induced by blasting in quarry sites. Nat. Resour. Res..

[37] Salazar L, Kogan F, Roytman L (2007). Use of remote sensing data for estimation of winter wheat yield in the United States. Int. J. Remote Sens..

[38] Shangguan W, Dai Y, Duan Q, Liu B, Yuan H (2014). A global soil data set for earth system modeling. J. Adv. Model. Earth Syst..

[39] Chen, T. and Guestrin, C. Xgboost: A scalable tree boosting system. In *Proc. of the 22nd acm sigkdd international conference on knowledge discovery and data mining* (2016).

[40] Breiman L (2001). Random forests. Mach. Learn..

[41] Magidi J (2021). Application of the random forest classifier to map irrigated areas using google earth engine. Remote Sens..

[42] Kouadri S (2021). Performance of machine learning methods in predicting water quality index based on irregular data set: Application on Illizi region (Algerian southeast). Appl. Water Sci..

[43] Trabelsi F, Bel Hadj Ali S (2022). Exploring machine learning models in predicting irrigation groundwater quality indices for effective decision making in Medjerda river Basin Tunisia. Sustainability.

[44] Ferreira LB, da Cunha FF (2020). Multi-step ahead forecasting of daily reference evapotranspiration using deep learning. Comput. Electron. Agric..

[45] Hochreiter SSJ (1997). Long short-term memory. Neural Comput..

[46] Wu Q, Lin H (2019). Daily urban air quality index forecasting based on variational mode decomposition, sample entropy and LSTM neural network. Sustain. Cities Soc..

[47] Zhu S (2020). Forecasting of water level in multiple temperate lakes using machine learning models. J. Hydrol..

[48] Kingma, D.P. and Ba, J. Adam: A method for stochastic optimization*.* Preprint at http://arXiv.org//1412.6980 (2014).

[49] Ferreira LB, da Cunha FF (2020). New approach to estimate daily reference evapotranspiration based on hourly temperature and relative humidity using machine learning and deep learning. Agric. Water Manag..

[50] Barzegar R, Aalami MT, Adamowski J (2020). Short-term water quality variable prediction using a hybrid CNN–LSTM deep learning model. Stoch. Environ. Res. Risk Assess..

[51] Zuo R, Xiong Y, Wang J, Carranza EJM (2019). Deep learning and its application in geochemical mapping. Earth-Sci. Rev..

[52] Glória A, Cardoso J, Sebastião P (2021). Sustainable irrigation system for farming supported by machine learning and real-time sensor data. Sensors.

[53] Aquil MAI, Ishak WHW (2021). Evaluation of scratch and pre-trained convolutional neural networks for the classification of Tomato plant diseases. IAES Int. J. Artif. Intell..

[54] Vicente-Serrano SM, Beguería S, López-Moreno JI (2010). A multiscalar drought index sensitive to global warming: The standardized precipitation evapotranspiration index. J. Clim..

[55] Potopová V (2016). Impact of agricultural drought on main crop yields in the Republic of Moldova. Int. J. Climatol..

[56] Lobell DB, Asner GP (2003). Climate and management contributions to recent trends in U. S. agricultural yields. Science.

[57] Wu H, Hubbard KG, Wilhite DA (2004). An agricultural drought risk-assessment model for corn and soybeans. Int. J. Climatol. J. R. Meteorol. Soc..

[58] Tigkas D, Vangelis H, Tsakiris G (2019). Drought characterisation based on an agriculture-oriented standardised precipitation index. Theor. Appl. Climatol..

[59] Ding Y, Wang W, Zhuang Q, Luo Y (2020). Adaptation of paddy rice in China to climate change: The effects of shifting sowing date on yield and irrigation water requirement. Agric. Water Manag..

[60] Wang J (2017). Growing water scarcity, food security and government responses in China. Glob. Food Secur..

[61] Chiu M-C, Wen C-Y, Hsu H-W, Wang W-C (2022). Key wastes selection and prediction improvement for biogas production through hybrid machine learning methods. Sustain. Energy Technol. Assess..

[62] Huang T, Liu T, Ai Y, Ren Z, Ou J, Li Y, Xu N (2023). Modelling the interface bond strength of corroded reinforced concrete using hybrid machine learning algorithms. J. Build. Eng..

[63] Wang P, Hu J, Chen W (2023). A hybrid machine learning model to optimize thermal comfort and carbon emissions of large-space public buildings. J. Clean. Prod..

[64] Sulaiman R (2024). Hybrid ensemble-based machine learning model for predicting phosphorus concentrations in hydroponic solution. Spectrochim. Acta A Mol. Biomol. Spectrosc..

[65] Huang L, Chen J, Tan X (2022). BP-ANN based bond strength prediction for FRP reinforced concrete at high temperature. Eng. Struct..

[66] Bai H, Tao F, Xiao D, Liu F, Zhang H (2016). Attribution of yield change for rice-wheat rotation system in China to climate change, cultivars and agronomic management in the past three decades. Clim. Change.

[67] Chen J, Theller L, Gitau MW, Engel BA, Harbor JM (2016). Urbanization impacts on surface runoff of the contiguous United States. J. Environ. Manag..

[68] Chen X, Chen S (2018). China feels the heat: negative impacts of high temperatures on China's rice sector. Aust. J. Agric. Resour. Econ..

[69] Maricle BR, Adler PB (2011). Effects of precipitation on photosynthesis and water potential in *Andropogon*
*gerardii* and *Schizachyrium*
*scoparium* in a southern mixed grass prairie. Environ. Exp. Bot..

[70] Liu L, Zhu Y, Tang L, Cao W, Wang E (2013). Impacts of climate changes, soil nutrients, variety types and management practices on rice yield in East China: A case study in the Taihu region. Field Crops Res..

[71] Wang W (2017). Bayesian multi-model projection of irrigation requirement and water use efficiency in three typical rice plantation region of China based on CMIP5. Agric. For. Meteorol..

[72] Moseley WG (2016). Agriculture on the brink: Climate change, labor and smallholder farming in Botswana. Land.

[73] Sala OE (2000). Global biodiversity scenarios for the year 2100. Science.

[74] Zare M (2016). Simulation of soil erosion under the influence of climate change scenarios. Environ. Earth Sci..

[75] Cao J (2021). Wheat yield predictions at a county and field scale with deep learning, machine learning, and google earth engine. Eur. J. Agron..

[76] Liu Y, Li N, Zhang Z, Huang C, Chen X, Wang F (2020). The central trend in crop yields under climate change in China: A systematic review. Sci. Total Environ..

[77] Boken VK, Shaykewich CF (2002). Improving an operational wheat yield model using phenological phase-based normalized difference vegetation index. Int. J. Remote Sens..

[78] Jiang H (2020). A deep learning approach to conflating heterogeneous geospatial data for corn yield estimation: A case study of the US Corn Belt at the county level. Glob. Change Biol..

[79] Alhaj Hamoud Y (2019). Effect of irrigation regimes and soil texture on the potassium utilization efficiency of rice. Agronomy.

[80] Dou F (2016). Soil texture and cultivar effects on rice (*Oryza*
*sativa*, L.) grain yield, yield components and water productivity in three water regimes. PLoS One.

[81] Rao PR (2013). Influence of boron on spikelet fertility under varied soil conditions in rice genotypes. J. Plant Nutr..

[82] Ma X (2013). Rice re-cultivation in southern China: An option for enhanced climate change resilience in rice production. J. Geogr. Sci..

[83] Yao L (2014). Current situation and prospect of rice water-saving irrigation technology in China. Chin. J. Ecol..

[84] Xie J, Luo J, Ma M, Xie J, Luo J, Ma M (1990). Potassium-supplying potential of different soils and the current potassium balance status in the farmland ecosystems in China. Proceedings of the International Symposium on Balanced Fertilization, Soil and Fertilizer Institute of the Chinese Academy of Agricultural Sciences.

[85] Bouman B, Tuong TP (2001). Field water management to save water and increase its productivity in irrigated lowland rice. Agric. Water Manag..

[86] Islam M (2004). Influence of cracking on rice seasons and irrigation in Bangladesh. J. Biol. Sci..

